# (*E*)-3-Chloro-*N*′-(2-fluoro­benzyl­idene)thio­phene-2-carbohydrazide

**DOI:** 10.1107/S1600536814011568

**Published:** 2014-06-04

**Authors:** Sadia Sultan, Muhammad Taha, Syed Adnan Ali Shah, Bohari M. Yamin, Hamizah Mohd Zaki

**Affiliations:** a Faculty of Pharmacy, University Teknologi Mara (UiTM), Puncak Alam Campus, 42300 Bandar Puncak Alam, Selangor D. E., Malaysia; b Atta-ur-Rahman Institute for Natural Product Discovery, Universiti Teknologi MARA (UiTM), Puncak Alam Campus, 42300 Bandar Puncak Alam, Selangor D. E., Malaysia; c Faculty of Applied Sciences, Universiti Teknologi MARA (UiTM), 40450 Shah Alam, Selangor D.E., Malaysia; d School of Chemical Sciences and Food Technology, Universiti Kebangsaan Malaysia, 43600 Bangi, Selangor D.E., Malaysia

## Abstract

The title compound, C_12_H_8_ClFN_2_OS, is a hydrazide derivative adopting an *E* conformation with an azomethine N=C double bond length of 1.272 (2) Å. The mol­ecular skeleton is approximately planar; the terminal five- and six-membered rings form a dihedral angle of 5.47 (9)°. In the crystal, mol­ecules are linked by N—H⋯O and C—H⋯O hydrogen bonds into zigzag chains propagating in [100].

## Related literature   

For the applications and biological activity of hydrazones, see: Taha *et al.* (2013 ▶); Musharraf *et al.* (2012 ▶); Melnyk *et al.* (2006 ▶); Terzioglu & Gursoy (2003 ▶). For the crystal structures of related compounds, see: Alanazi *et al.* (2012*a* ▶,*b* ▶). 
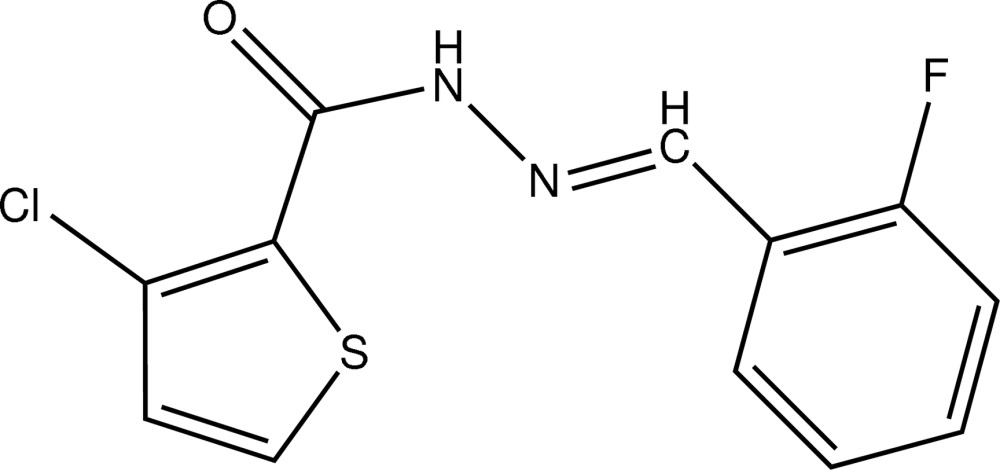


## Experimental   

### 

#### Crystal data  

C_12_H_8_ClFN_2_OS
*M* *_r_* = 282.71
Orthorhombic, 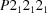

*a* = 5.6833 (3) Å
*b* = 13.0817 (6) Å
*c* = 16.4001 (8) Å
*V* = 1219.30 (10) Å^3^
*Z* = 4
Mo *K*α radiation
μ = 0.48 mm^−1^
*T* = 302 K
0.55 × 0.46 × 0.03 mm

#### Data collection  

Bruker SMART APEX CCD area-detector diffractometer
Absorption correction: multi-scan (*SADABS*; Bruker, 2000 ▶) *T*_min_ = 0.776, *T*_max_ = 0.985
47474 measured reflections
2255 independent reflections
2210 reflections with *I* > 2σ(*I*)
*R*_int_ = 0.028

#### Refinement  

*R*[*F* ^2^ > 2σ(*F* ^2^)] = 0.023
*wR*(*F* ^2^) = 0.065
*S* = 1.09
2255 reflections
168 parameters
H atoms treated by a mixture of independent and constrained refinement
Δρ_max_ = 0.15 e Å^−3^
Δρ_min_ = −0.12 e Å^−3^
Absolute structure: Flack (1983 ▶), 916 Friedel pairs
Absolute structure parameter: 0.02 (5)

### 

Data collection: *SMART* (Bruker, 2000 ▶); cell refinement: *SAINT* (Bruker, 2000 ▶); data reduction: *SAINT*; program(s) used to solve structure: *SHELXTL* (Sheldrick, 2008 ▶); program(s) used to refine structure: *SHELXTL*; molecular graphics: *SHELXTL*; software used to prepare material for publication: *SHELXTL* and *PLATON* (Spek, 2009 ▶).

## Supplementary Material

Crystal structure: contains datablock(s) global, I. DOI: 10.1107/S1600536814011568/cv5453sup1.cif

Structure factors: contains datablock(s) I. DOI: 10.1107/S1600536814011568/cv5453Isup2.hkl

Supporting information file. DOI: 10.1107/S1600536814011568/cv5453Isup3.cml

CCDC reference: 1003818

Additional supporting information:  crystallographic information; 3D view; checkCIF report

## Figures and Tables

**Table 1:** Hydrogen-bond geometry (Å, °)

*D*—H⋯*A*	*D*—H	H⋯*A*	*D*⋯*A*	*D*—H⋯*A*
N1—H1*A*⋯O1^i^	0.86	2.12	2.9552 (18)	163
C7—H7*A*⋯O1^i^	0.93	2.41	3.2268 (19)	147

Symmetry code: (i) 
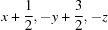
.

**Fig. 1.**
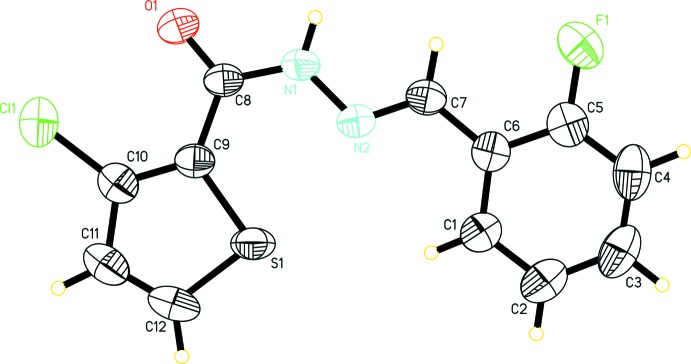
The molecular structure of (I) with displacement ellipsoids drawn at the 50% probability level.

**Fig. 2.**
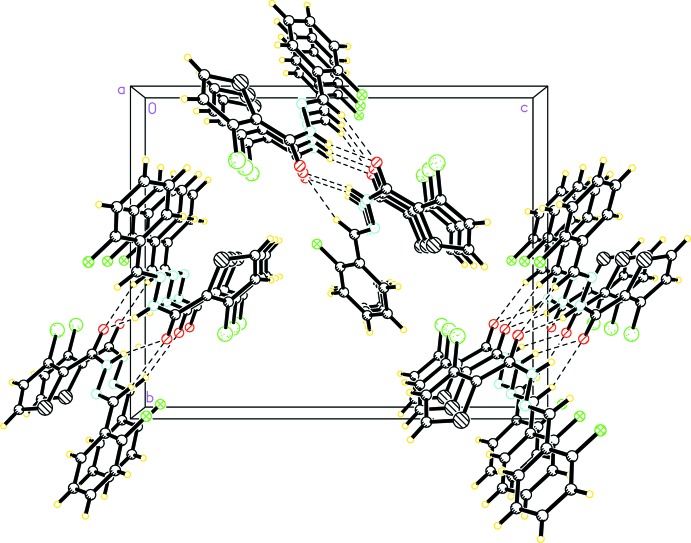
A portion of the crystal packing viewed down the a axis. Dashed lines denote hydrogen bonds.

## supplementary crystallographic information

### S1. Comment

Hydrazone derivatives are known as good ligands for complexation reactions. They have also displayed a wide spectrum of biological activities including antileishamanial (Taha *et al.*, 2013), antimalarial (Melnyk *et al.*, 2006) and anti-cancer (Terzioglu *et al.*, 2003) properties. Recently the hydrazones are reported to be used as UV-LDI Matrices for measuring the mass of macromolecules (Musharraf *et al.*, 2012) .

The title compound, (I) (Fig. 1), is similar to that of previously reported N'-[(1E)-(2,6-difluorophenyl)methylidene]thiophene-2-carbohydrazide (Alanazi *et al.*, 2012*a*) and N'-[(1E)-(4-fluorophenyl)methylidene]- thiophene-2-carbohydrazide (Alanazi *et al.*, 2012*b*) except the thiophene ring is substituted with fluorine atom. The whole molecule is appearently planar with maximum deviation of 0.181 (1)Å for F1 atom from the least square plane. The chlorothiophenecarbonyl O1/C8/S1/(C9-C12)/Cl fragment is trans to the fluorobenzyl, F1/(C1-C7), group across the N1-N2 bond. The bond lengths and angles in (I) are normal and comparable to those in the analogs (Alanazi *et al.*, 2012*a*,*b*). The crystal is stablized by N—H···O and C—H···O intermolecular hydrogen bonds (Table 1) to form zigzag chains of molecules extended along the a axis (Fig. 2).

### S2. Experimental

The title compound (I) was synthesized by refluxing in methanol a mixture (0.352 g, 2 mmol) of 3-chlorothiophene- 2-carbohydrazide and (0.248 g, 2 mmol) of 2 florobenzaldehyde along with a catalytical amount of acetic acid for 3 h. The progress of reaction was monitored by TLC. After completion of reaction, the solvent was evaporated by vacuum to afford crude material which was purified by repeated recrystallized in methanol to obtain needle like crytals (0.495 g, ° yielded 88). All chemicals (methyl 3-chlorothiophene-2-carboxylate 99%,2-florobenzaldehyde 98%) were purchased from sigma Aldrich.

### S3. Refinement

All H atoms except H12A were positioned geometrically (C—H = 0.93 Å and N—H 0.86 Å) and constrained to ride on their parent atoms with *U*_iso_(H) = 1.2*U*_eq_(C, N). Atom H12A attached to C12 was located on a Fourier map and isotropically refined.

### Crystal data

**Table:**

C_12_H_8_ClFN_2_OS	*F*(000) = 576
*M**_r_* = 282.71	*D*_x_ = 1.540 Mg m^−^^3^
Orthorhombic, *P*2_1_2_1_2_1_	Mo *K*α radiation, λ = 0.71073 Å
Hall symbol: P 2ac 2ab	Cell parameters from 9699 reflections
*a* = 5.6833 (3) Å	θ = 3.1–25.5°
*b* = 13.0817 (6) Å	µ = 0.48 mm^−^^1^
*c* = 16.4001 (8) Å	*T* = 302 K
*V* = 1219.30 (10) Å^3^	Slab, colourless
*Z* = 4	0.55 × 0.46 × 0.03 mm

### Data collection

**Table:**

Bruker SMART APEX CCD area-detector diffractometer	2255 independent reflections
Radiation source: fine-focus sealed tube	2210 reflections with *I* > 2σ(*I*)
Graphite monochromator	*R*_int_ = 0.028
Detector resolution: 83.66 pixels mm^-1^	θ_max_ = 25.5°, θ_min_ = 3.1°
ω scan	*h* = −6→6
Absorption correction: multi-scan (*SADABS*; Bruker, 2000)	*k* = −15→15
*T*_min_ = 0.776, *T*_max_ = 0.985	*l* = −19→19
47474 measured reflections	

### Refinement

**Table:**

Refinement on *F*^2^	Hydrogen site location: inferred from neighbouring sites
Least-squares matrix: full	H atoms treated by a mixture of independent and constrained refinement
*R*[*F*^2^ > 2σ(*F*^2^)] = 0.023	*w* = 1/[σ^2^(*F*_o_^2^) + (0.0388*P*)^2^ + 0.1642*P*] where *P* = (*F*_o_^2^ + 2*F*_c_^2^)/3
*wR*(*F*^2^) = 0.065	(Δ/σ)_max_ < 0.001
*S* = 1.09	Δρ_max_ = 0.15 e Å^−^^3^
2255 reflections	Δρ_min_ = −0.12 e Å^−^^3^
168 parameters	Extinction correction: *SHELXTL* (Sheldrick, 2008), Fc^*^=kFc[1+0.001xFc^2^λ^3^/sin(2θ)]^-1/4^
0 restraints	Extinction coefficient: 0.021 (2)
Primary atom site location: structure-invariant direct methods	Absolute structure: Flack (1983), 916 Friedel pairs
Secondary atom site location: difference Fourier map	Absolute structure parameter: 0.02 (5)

### Special details

**Table:**

Geometry. All e.s.d.'s (except the e.s.d. in the dihedral angle between two l.s. planes) are estimated using the full covariance matrix. The cell e.s.d.'s are taken into account individually in the estimation of e.s.d.'s in distances, angles and torsion angles; correlations between e.s.d.'s in cell parameters are only used when they are defined by crystal symmetry. An approximate (isotropic) treatment of cell e.s.d.'s is used for estimating e.s.d.'s involving l.s. planes.
Refinement. Refinement of *F*^2^ against ALL reflections. The weighted *R*-factor *wR* and goodness of fit *S* are based on *F*^2^, conventional *R*-factors *R* are based on *F*, with *F* set to zero for negative *F*^2^. The threshold expression of *F*^2^ > σ(*F*^2^) is used only for calculating *R*-factors(gt) *etc*. and is not relevant to the choice of reflections for refinement. *R*-factors based on *F*^2^ are statistically about twice as large as those based on *F*, and *R*- factors based on ALL data will be even larger.

### Fractional atomic coordinates and isotropic or equivalent isotropic displacement parameters (Å^2^)

**Table:**

	*x*	*y*	*z*	*U*_iso_*/*U*_eq_	
S1	0.17368 (8)	0.52165 (3)	0.22226 (3)	0.05051 (13)	
Cl1	−0.35177 (8)	0.75148 (4)	0.21939 (3)	0.06171 (15)	
F1	0.9968 (2)	0.52634 (9)	−0.05506 (7)	0.0641 (3)	
O1	−0.0135 (3)	0.75985 (10)	0.08685 (8)	0.0592 (3)	
N1	0.2895 (2)	0.65589 (9)	0.06578 (7)	0.0424 (3)	
H1A	0.3269	0.6912	0.0235	0.051*	
N2	0.4234 (2)	0.57273 (10)	0.08483 (8)	0.0402 (3)	
C1	0.7057 (3)	0.39194 (12)	0.10909 (10)	0.0498 (4)	
H1B	0.5728	0.3967	0.1420	0.060*	
C2	0.8577 (4)	0.31121 (13)	0.11894 (13)	0.0602 (5)	
H2B	0.8273	0.2620	0.1584	0.072*	
C3	1.0558 (4)	0.30280 (15)	0.07046 (15)	0.0652 (5)	
H3A	1.1577	0.2479	0.0776	0.078*	
C4	1.1029 (3)	0.37504 (14)	0.01183 (13)	0.0618 (5)	
H4A	1.2357	0.3698	−0.0210	0.074*	
C5	0.9492 (3)	0.45490 (13)	0.00305 (10)	0.0484 (4)	
C6	0.7486 (3)	0.46727 (12)	0.04998 (9)	0.0428 (3)	
C7	0.5958 (3)	0.55497 (12)	0.03748 (9)	0.0430 (4)	
H7A	0.6247	0.5989	−0.0059	0.052*	
C8	0.1010 (3)	0.68555 (11)	0.11008 (9)	0.0396 (3)	
C9	0.0325 (3)	0.62923 (11)	0.18418 (9)	0.0392 (3)	
C10	−0.1608 (3)	0.65037 (12)	0.23145 (10)	0.0459 (3)	
C11	−0.1957 (4)	0.58121 (15)	0.29608 (10)	0.0599 (5)	
H11A	−0.3197	0.5852	0.3330	0.072*	
C12	−0.0282 (4)	0.50870 (17)	0.29804 (12)	0.0654 (5)	
H12A	−0.005 (5)	0.4586 (17)	0.3352 (16)	0.086 (7)*	

### Atomic displacement parameters (Å^2^)

**Table:**

	*U*^11^	*U*^22^	*U*^33^	*U*^12^	*U*^13^	*U*^23^
S1	0.0573 (2)	0.0498 (2)	0.0445 (2)	0.00003 (18)	−0.00151 (19)	0.01644 (17)
Cl1	0.0550 (2)	0.0632 (3)	0.0670 (3)	0.0061 (2)	0.0052 (2)	−0.0115 (2)
F1	0.0649 (6)	0.0732 (7)	0.0542 (6)	−0.0086 (6)	0.0104 (5)	−0.0038 (5)
O1	0.0655 (8)	0.0571 (7)	0.0551 (7)	0.0185 (6)	0.0050 (6)	0.0202 (6)
N1	0.0472 (7)	0.0429 (6)	0.0370 (6)	0.0018 (6)	0.0005 (6)	0.0104 (5)
N2	0.0439 (7)	0.0378 (6)	0.0389 (6)	−0.0010 (5)	−0.0040 (5)	0.0050 (5)
C1	0.0503 (9)	0.0459 (8)	0.0533 (9)	0.0002 (7)	−0.0053 (8)	0.0012 (7)
C2	0.0631 (11)	0.0470 (9)	0.0706 (11)	0.0036 (8)	−0.0161 (10)	0.0002 (8)
C3	0.0560 (11)	0.0507 (10)	0.0889 (15)	0.0117 (8)	−0.0153 (11)	−0.0148 (10)
C4	0.0455 (10)	0.0655 (11)	0.0746 (12)	0.0037 (8)	−0.0020 (9)	−0.0256 (10)
C5	0.0493 (9)	0.0503 (9)	0.0457 (8)	−0.0072 (7)	−0.0041 (7)	−0.0125 (7)
C6	0.0420 (8)	0.0443 (8)	0.0421 (7)	−0.0034 (6)	−0.0059 (6)	−0.0059 (6)
C7	0.0474 (8)	0.0436 (8)	0.0380 (7)	−0.0045 (6)	−0.0026 (6)	0.0026 (6)
C8	0.0437 (8)	0.0395 (7)	0.0355 (7)	−0.0019 (6)	−0.0057 (6)	0.0051 (6)
C9	0.0430 (8)	0.0401 (7)	0.0345 (7)	−0.0050 (6)	−0.0064 (6)	0.0026 (6)
C10	0.0476 (8)	0.0497 (8)	0.0406 (8)	−0.0089 (7)	−0.0018 (7)	−0.0049 (6)
C11	0.0673 (11)	0.0673 (11)	0.0452 (9)	−0.0139 (10)	0.0113 (8)	0.0027 (8)
C12	0.0806 (14)	0.0708 (12)	0.0447 (9)	−0.0108 (11)	0.0056 (9)	0.0191 (9)

### Geometric parameters (Å, º)

**Table:**

S1—C12	1.700 (2)	C3—C4	1.375 (3)
S1—C9	1.7362 (15)	C3—H3A	0.9300
Cl1—C10	1.7224 (18)	C4—C5	1.369 (3)
F1—C5	1.362 (2)	C4—H4A	0.9300
O1—C8	1.2304 (19)	C5—C6	1.385 (2)
N1—C8	1.351 (2)	C6—C7	1.453 (2)
N1—N2	1.3639 (17)	C7—H7A	0.9300
N1—H1A	0.8600	C8—C9	1.474 (2)
N2—C7	1.272 (2)	C9—C10	1.373 (2)
C1—C2	1.374 (2)	C10—C11	1.408 (2)
C1—C6	1.404 (2)	C11—C12	1.344 (3)
C1—H1B	0.9300	C11—H11A	0.9300
C2—C3	1.382 (3)	C12—H12A	0.90 (2)
C2—H2B	0.9300		
			
C12—S1—C9	91.82 (10)	C5—C6—C7	120.37 (15)
C8—N1—N2	123.24 (12)	C1—C6—C7	123.21 (15)
C8—N1—H1A	118.4	N2—C7—C6	121.23 (14)
N2—N1—H1A	118.4	N2—C7—H7A	119.4
C7—N2—N1	115.83 (13)	C6—C7—H7A	119.4
C2—C1—C6	120.76 (17)	O1—C8—N1	118.68 (14)
C2—C1—H1B	119.6	O1—C8—C9	120.69 (14)
C6—C1—H1B	119.6	N1—C8—C9	120.63 (13)
C1—C2—C3	120.37 (18)	C10—C9—C8	125.21 (14)
C1—C2—H2B	119.8	C10—C9—S1	109.27 (11)
C3—C2—H2B	119.8	C8—C9—S1	125.43 (12)
C4—C3—C2	120.41 (18)	C9—C10—C11	114.11 (16)
C4—C3—H3A	119.8	C9—C10—Cl1	126.46 (13)
C2—C3—H3A	119.8	C11—C10—Cl1	119.42 (14)
C5—C4—C3	118.29 (18)	C12—C11—C10	111.83 (17)
C5—C4—H4A	120.9	C12—C11—H11A	124.1
C3—C4—H4A	120.9	C10—C11—H11A	124.1
F1—C5—C4	118.06 (17)	C11—C12—S1	112.96 (14)
F1—C5—C6	118.18 (16)	C11—C12—H12A	129.0 (18)
C4—C5—C6	123.76 (18)	S1—C12—H12A	117.9 (18)
C5—C6—C1	116.41 (16)		
			
C8—N1—N2—C7	−179.62 (14)	N2—N1—C8—C9	1.4 (2)
C6—C1—C2—C3	−0.2 (3)	O1—C8—C9—C10	2.1 (2)
C1—C2—C3—C4	0.0 (3)	N1—C8—C9—C10	−177.17 (14)
C2—C3—C4—C5	0.1 (3)	O1—C8—C9—S1	178.35 (13)
C3—C4—C5—F1	179.92 (17)	N1—C8—C9—S1	−0.9 (2)
C3—C4—C5—C6	0.1 (3)	C12—S1—C9—C10	0.39 (13)
F1—C5—C6—C1	179.91 (14)	C12—S1—C9—C8	−176.37 (14)
C4—C5—C6—C1	−0.2 (2)	C8—C9—C10—C11	176.15 (14)
F1—C5—C6—C7	−0.6 (2)	S1—C9—C10—C11	−0.62 (18)
C4—C5—C6—C7	179.24 (15)	C8—C9—C10—Cl1	−5.0 (2)
C2—C1—C6—C5	0.3 (2)	S1—C9—C10—Cl1	178.22 (10)
C2—C1—C6—C7	−179.19 (15)	C9—C10—C11—C12	0.6 (2)
N1—N2—C7—C6	−178.51 (13)	Cl1—C10—C11—C12	−178.35 (14)
C5—C6—C7—N2	−173.37 (14)	C10—C11—C12—S1	−0.3 (2)
C1—C6—C7—N2	6.1 (2)	C9—S1—C12—C11	−0.07 (17)
N2—N1—C8—O1	−177.85 (14)		

### Hydrogen-bond geometry (Å, º)

**Table:**

*D*—H···*A*	*D*—H	H···*A*	*D*···*A*	*D*—H···*A*
N1—H1*A*···O1^i^	0.86	2.12	2.9552 (18)	163
C7—H7*A*···O1^i^	0.93	2.41	3.2268 (19)	147

Symmetry code: (i) *x*+1/2, −*y*+3/2, −*z*.
